# Stiff Landings, Core Stability, and Dynamic Knee Valgus: A Systematic Review on Documented Anterior Cruciate Ligament Ruptures in Male and Female Athletes

**DOI:** 10.3390/ijerph18073826

**Published:** 2021-04-06

**Authors:** Joseph Larwa, Conrad Stoy, Ross S. Chafetz, Michael Boniello, Corinna Franklin

**Affiliations:** 1 Drexel University College of Medicine, Philadelphia, PA 19129, USA; joseph.larwa@gmail.com (J.L.); cs3623@drexel.edu (C.S.); 2 Shriners Hospital for Children—Philadelphia, Philadelphia, PA 19140, USA; rchafetz@shrinenet.org (R.S.C.); boniello-michael@cooperhealth.edu (M.B.)

**Keywords:** ACL, knee, valgus, abduction, adduction, flexion, trunk, core

## Abstract

Anterior cruciate ligament (ACL) injuries are the most common ligament injury of the knee, accounting for between 100,000 and 200,000 injuries among athletes per year. ACL injuries occur via contact and non-contact mechanisms, with the former being more common in males and the later being more common in females. These injuries typically require surgical repair and have relatively high re-rupture rates, resulting in a significant psychological burden for these individuals and long rehabilitation times. Numerous studies have attempted to determine risk factors for ACL rupture, including hormonal, biomechanical, and sport- and gender-specific factors. However, the incidence of ACL injuries continues to rise. Therefore, we performed a systematic review analyzing both ACL injury video analysis studies and studies on athletes who were pre-screened with eventual ACL injury. We investigated biomechanical mechanisms contributing to ACL injury and considered male and female differences. Factors such as hip angle and strength, knee movement, trunk stability, and ankle motion were considered to give a comprehensive, joint by joint analysis of injury risk and possible roles of prevention. Our review demonstrated that poor core stability, landing with heel strike, weak hip abduction strength, and increased knee valgus may contribute to increased ACL injury risk in young athletes.

## 1. Introduction

Anterior cruciate ligament (ACL) injuries are the most common traumatic knee ligament injuries, frequently affecting young athletes [1,2]. They typically occur via non-contact, low-energy mechanisms and require significant intensive rehabilitation prior to resumption of athletics [3,4]. Decelerating, cutting, and rotational moments performed by athletes, especially during landing, are the most common mechanisms for ACL rupture. [4]. Current evidence estimates the average incidence of ACL rupture to be approximately 1 in 3500 across athlete populations [1,2]. While male football players appear to have the highest incidence of ACL injuries, these are typically due to direct contact mechanisms [5]. Females, however, are at a higher risk of ACL injury from non-contact mechanisms. Additionally, female injury rates per exposure are higher when compared to males, potentially due to anatomic differences of the lower kinematic chain [6]. Females who participate in gymnastics, soccer, or basketball appear to be at the highest risk of experiencing a non-contact ACL injury [5,6].

Video analysis of ACL injuries and pre-screening athletes who go on to experience an ACL injury are vital for understanding the biomechanics that predispose athletes to sustaining these injuries. Additionally, these studies can help guide targeted training programs to help improve athletic performance and decrease the risk of lower extremity injuries [7,8,9,10,11]. Various factors are thought to contribute to ACL injury incidence. Perhaps the most widely considered risk factor is dynamic knee valgus, which places significant tensile forces on the ACL especially during landing and cutting [12,13,14]. Knee valgus may occur secondary to many factors, including but not limited to weak hip abductor strength, poor hip musculature control, increased femoral anteversion/medial tibial torsion, wider pelvis, increase midfoot mobility, and larger q-angle. Anatomically speaking, females have wider hips, which predisposes them to larger q-angles and subsequent higher risk of ACL injury than males [15,16]. Females also typically do not generate as much force as male counterparts in the hip abductors, thus potentially subjecting them to dynamic knee valgus moments [17].

Other potential risk factors that have been theorized for ACL injury include poor postural control and more upright landings. Poor postural control may result in the inability to appropriately respond to perturbations, which increases the stabilizing requirement of the ACL during movements and play [18,19,20]. More upright landings prevent the hamstrings from maximally restraining the anterior translation of the tibia on the femur, thus theoretically placing the ACL under increased stress with load acceptance [21,22,23]. 

Due to the large volume of research on ACL injuries and numerous injury theories, a comprehensive review of the relationship between kinematics, kinetics, and risk of injury to the ACL is needed to summarize key findings. This is especially true when considering the role that sex differences contribute to this presumed biomechanical risk. Therefore, the purpose of this systematic review was to assess biomechanical factors that influence risk of ACL rupture in males and females and to analyze if differences exist for those with a documented ACL rupture. We also assessed the kinematic profile that increases ACL injury risk for both sexes. Video analysis studies were utilized in order to analyze real life injury situations as well as biomechanical pre-screened laboratory data to create the most encompassing review of current literature. 

## 2. Materials and Methods

*Literature Search:* A systematic review of the literature was performed from inception through April 1, 2020 using Web of Science, CINHAL, PubMed, and Cochrane Collaboration. The search criteria we used were as follows:

ACL Injur* OR Anterior Cruciate Ligament Injur* AND Video Analysis OR Motion Analysis OR Movie OR Screen

The reference list from each included article was analyzed for additional articles. However, all included articles were found from the original search criteria.

*Selection Criteria:* All articles populated by the search criteria were imported into Rayyan, a web-based application that facilitates systematic review article screening, and an initial screening was performed by one author (J.L.). Articles were initially screened by the title and abstract and screened for more thorough analysis with the full-text article. Another author (M.B.) was consulted for deliberation of articles that were difficult to assess, with the most senior writers (C.F. and R.C.) making the final decision for inclusion. Two article styles, pre-screened (PS) players and video analysis (VA) were included. Pre-screening studies include those that take baseline measurements in a laboratory setting (i.e., one-legged box jumps, mean knee flexion at initial impact, etc.) and longitudinally follow athletes to assess factors that may increase athlete injury risk. Video analysis studies largely focus on documented scenarios where injury was known to have occurred and utilize computer programs to measure joint angles and follow bodily motion throughout the movement. Once such measurements are taken, values are either compared to similar, non-injury situations or known mean values to assess how injury situations typically differ from non-injury situations. Articles were included if there was a documentation of primary ACL rupture and mention of sex. In addition, articles were included if there was kinematic assessment prior to injury documented for PS and if there was a kinematic analysis of the video in which the ACL injury occurred for VA. Articles were excluded if subjects had a primary ACL reconstruction prior to the study, study participants did not have a primary ACL injury, and if they were written in a language other than English or were review papers.

*Please see Supplementary Materials for a PRISMA flow diagram of the search.*

*Study Quality Assessment:* Risk of bias assessment was performed using Melnyk et al.’s hierarchy of evidence [24]. This hierarchy is commonly used to assess study quality in the health care field. Studies are graded on a seven-tiered scale with lower numerical values representing higher levels of evidence.

*Data Extraction and Analysis:* Full text of the articles that met inclusion criteria were retrieved and further assessed. Outcome data were extracted independently by one reviewer (J.L.) and subsequently verified by the other reviewers. Additional data was collected from one paper by directly contacting the corresponding author. Data extracted for VA and PS can be found in Table 1. If data were presented that could not directly be compared among the remaining papers, every effort was made to convert the data into compatible formats. This was performed for one study in this review [11]. Dingenen et al. defined hip flexion as “in the sagittal plane … the angle between the line formed by the acromioclavicular joint and the greater trochanter, and a second line connecting the greater trochanter to the lateral femoral epicondyle.” In all other included studies, hip flexion was defined as the inverse of the aforementioned measurement. Therefore, hip flexion from the Dingenen et al. study was subtracted from 180 which allowed direct comparison across all studies. Data collected was directed at known factors that play roles in ACL injury and/or stabilization such as hip flexion, hip abduction, knee flexion, knee abduction, ankle motion, and trunk angle/lean.

## 3. Results

After duplicates were removed, our search criteria yielded 1880 total articles. After thorough analysis of each of these articles, our review featured eighteen studies that met inclusion criteria. Of those included, nine were video analysis studies and nine were pre-screening studies (Table 1). The majority of injuries in the video analysis studies occurred while athletes were on offense (47%), had the ball (29%) or were performing a cutting motion (20%). Additionally, injuries observed via VA reviewed in this paper featured mostly basketball (47%), handball (24%), and rugby (13%) players, with soccer (12%), American football (3%), and gymnastics (1%) being less represented. Injuries seen in pre-screening studies mostly occurred while athletes were participating in floorball (34%), basketball (33%), handball (20%), American football (12%), and volleyball (1%).

*Hip Flexion:* Hip flexion was considered in nine studies (2 PS, 7 VA) [11,15,18,25,26,27,28,29,31]. Higher degrees of hip flexion at initial contact in female patients sustaining ACL injuries versus control subjects were found in two out of five studies (Table 2) [15,25,26,27,31]. Boden et al. found that although degree of hip flexion was significantly higher during early stages of initial contact for both male and females who sustained ACL injury, it only remained significantly different through the end of the landing phase in female athletes [25]. They also reported a trend towards higher degree of peak hip flexion in injured participants versus non-injured (55.2° versus 41.2°, respectively) (Table 2) [25]. In addition, Koga et al. reported that mean hip flexion degree (51°) was sustained through the early stages of initial contact in injured athletes versus uninjured [26]. Krosshaug et al. found that mean hip flexion angles at initial contact (27° vs. 19°, *p* = 0.043) and 50 ms into loading (33° vs. 22°, *p* = 0.020) were significantly higher in females compared to males who suffered an ACL injury [15].

While some studies reported high degrees of hip flexion in both males and females during injury (≥40°) [15,25] Incorrect order of references, you skipped reference [31]. Please revise so all references appear in numerical order [29,31], other studies contradicted this finding [11,27]. Leppänen et al. found no significant relationship for pre-injury peak hip flexion moment between injured and non-injured groups [31]. Despite this, they found that a stiffer landing (i.e., less hip flexion) was significantly associated with ACL injury (HR for each 10° increase in hip ROM, 0.61 [95% CI, 0.38–0.99]; *p* < 0.05) [31]. Montgomery et al. found no significant difference between the degree of hip flexion at initial contact and ACL injury susceptibility in males [27]. In these instances, uninjured male patients had a trend toward less hip flexion than ACL injured males at initial contact (43° ± 24° versus 26° ± 15°, *p* = 0.26) (Table 2) [27]. Digenen et al found no significant difference between pre-screened peak hip flexion angle for ACL injured subjects when compared to their uninjured counterparts (48.2° ± 14.1° versus 50.7° ± 10.5°, *p* = 0.585) [11]. Hewett et al. showed that peak external hip flexion moment was greater during ACL injury versus uninjured controls (147 ± 33 N·m versus 106 ± 45 N·m; *p* < 0.01) (Table 2) [18]. 

*Hip Abduction:* Hip abduction during ACL injury was directly evaluated in four VA studies [15,25,26,29]. Koga et al. analyzed ten cases of ACL injuries in female basketball players, all of whom were on offense at the time of injury with seven performing a cutting motion [26]. The mean hip abduction angle in these patients was 21° at initial contact which decreased by 6° through early loading [26]. Boden et al. specifically analyzed differences in hip abduction during the first 40 ms after initial contact, within which ACL injury likely occurred, between injured and non-injured males and females. They found no significant difference in mean hip abduction between injured and uninjured subjects, although injured subjects tended towards overall more hip abduction at the time of injury with a mean difference of 3.7° ± 11.7° (Table 2) [25]. They also found no significant sex differences regarding mean hip abduction between injured and non-injured athletes [25]. Another study reported high degrees of hip abduction (<20°) at initial contact was found to contribute to ACL injury in cutting and jumping/landing maneuvers [29]. Similarly, in a video analysis study on male American National Football League players, Johnston et al. reported that the most common position of the hip was abducted during initial contact for those sustaining an ACL injury in 43 out of 50 cases reviewed [35]. Krosshaug et al. found no significant difference between ACL-injured male and female athletes regarding hip abduction angle at landing and 50 ms after landing [15]. 

Khayambashi et al. compared isometric hip abduction strength via handheld dynamometer to assess its relation to ACL injury risk in both male and female athletes [17]. This group showed that non-injured athletes had significantly greater external hip rotation strength (*p* = 0.003) and hip abduction strength (*p* < 0.001) versus injured athletes. They suggested that there was a statistically significant risk of ACL injury corresponding to decreased hip abduction strength. Additionally, they found that male participants had greater hip abduction strength as a percentage of their total body weight when compared to their female peers [17].

*Knee Flexion:* Knee flexion degree was analyzed in seven VA studies and one PS study (Table 3) [12,15,18,25,27,28,30,32]. All studies included male and female subjects except Montgomery et al. which included only male subjects. In this study, they found that less knee flexion angle (≤20°) at initial contact was associated with increased risk of non-contact ACL injuries in males (Table 3) [27]. In their PS study, Leppänen et al. found no statistically significant difference in knee flexion angle at initial contact between injured and non-injured athletes [32]. They did report that a higher peak knee flexion angle over the duration of a vertical drop-jump test decreased the risk of subsequent ACL injury [32]. Additionally, they found that an increased knee flexion moment contributed to an increased risk of ACL injury with a hazard rate of 1.21 for each 10-N⋅m increase in knee moment (95% CI, 1.04–1.40, *p* < 0.07) [32]. They, as well as Olsen et al., suggested that reduced knee flexion coupled with greater knee moment (i.e., stiff landings) may be a risk factor for increased ACL injury [30,32]. While three studies found no difference in mean knee flexion at initial contact in movements that resulted in ACL injury [12,25,32], two studies found peak degree of knee flexion angle to be smaller in those that suffered ACL injury than their non-injured counterparts (Table 3) [18,25]. Interestingly, Krosshaug et al. found that mean knee flexion angle at initial contact (15° vs. 9°, *p* = 0.034) and 50 ms into loading (27° vs. 19°, *p* = 0.042) was significantly higher in ACL-injured females compared to ACL-injured male athletes in this cohort [15]. As reported previously, the same study showed hip flexion angles at initial contact and 50 ms into loading were higher in females signifying that despite less stiff landings, females were at a higher risk. 

*Knee Abduction:* Four PS studies analyzed knee abduction angle at initial contact in reference to risk of ACL injury (Table 3) [12,18,32,33]. Of these, two reported no statistically significant association between knee abduction at initial contact and ACL injury (Table 3) [32,33]. Conversely, Hewett et al. found that injured females had 8.4 degrees greater knee abduction at initial contact and 7.6 degrees higher at maximum versus female controls (*p* < 0.01) [18]. The group also demonstrated that injured females had statistically significant increases in stance phase peak knee abduction moment versus control (−45.3 ± 28.5 N⋅m vs. −18.4 ± 15.6 N⋅m, *p* < 0.001) (Table 3) [18]. Similarly, Numata et al. and Olsen et al. reinforced these findings by demonstrating that dynamic knee valgus is a potential risk factor for ACL injury in female athletes [30,33]. Specifically, Numata et al. found that dynamic knee valgus was significantly higher at initial contact in injured athletes versus non-injured at initial hallux-ground contact (2.1 ± 2.4 vs. 0.4 ± 2.2 cm, *p* = 0.006) (Table 3) [33]. Krosshaug et al. conducted a longitudinal study of elite female soccer and handball players via motion analysis of a drop-jump landing [12]. Unlike Hewett et al., they found no difference in knee abduction angle or moment at initial contact [12]. Although knee abduction angle and moments were not increased, they did find that greater medial knee displacement during the contact phase of the drop-jump increased injury risk by approximately 40% for a 1.2 cm increase in medial knee displacement (−2.2 ± 4.7° vs. 1.7 ± 4.1, *p* = 0.51) (Table 3) [12]. 

Three VA studies were included that analyzed knee abduction [15,25,33]. One study found no associated risk in peak knee abduction angle at initial contact in regards to ACL injury [25]. In this study, as the movement progressed, knee abduction angle remained more consistent in non-injured groups versus injured groups, with injured subjects progressing towards more significant knee abduction [25]. This progressive difference in knee abduction became significant at the third frame out of five (37.7 ± 21.0 degrees and 9.0 ± 17.1 degrees for injury versus controls) (Table 3) [25]. Additionally, this same study found that by the fifth frame of the sequence, females that sustained ACL injury had significantly higher knee abduction than their male injury counterparts [25]. Two different VA studies found that female ACL-injured athletes had significantly greater knee abduction on landing versus male ACL-injured athletes [15,33]. Krosshaug et al. found no significant gender differences in knee abduction at initial contact but did find an increased risk of valgus knee collapse (8° vs. 4°, *p* = 0.018) during load acceptance in female athletes when compared to their male counterparts (RR = 5.3; *p* = 0.002) [15]. These findings suggest that dynamic knee valgus may play a role in non-contact ACL injury and for the disparities among risk for female and male athletes.

*Ankle Motion:* Four VA studies [25,26,27,29] and one PS study [36] included analysis of ankle motion in consideration of ACL injury risk factors (Table 4). Boden et al. reported after jumping or stepping when initially contacting the ground the that mean ankle plantarflexion was significantly less in injured subjects at initial contact versus controls (10.7° ± 9.6° versus 22.9° ± 10.1°) (Table 4) [25]. They also demonstrated that injured athletes did not transition into dorsiflexion to the same extent as the controls. By foot-flat, control athletes had achieved 18.2° of dorsiflexion whereas injured athletes only had 9.4° (*p* < 0.0001). [25]. They suggest that the lack of ankle range at landing shows abnormal absorption of ground reaction forces by the gastrocnemius-soleus complex (Table 4) [25]. No significant difference was found between male and female subjects [25]. Similar findings were reported by Koga et al., who showed that all players in their case series of ten subjects landed in a heel-strike position with a mean dorsiflexion of 2° at initial contact and transferred to a flat-foot position over the next 20 ms by increasing the plantarflexion angle by an average of 12° [26]. Montgomery et al. also showed that male ACL injuries tended to occur more with heel strike at initial contact with median ankle plantar flexion being 10° in injury cases and 0° in non-injury cases [27]. Additionally, while their findings were not conclusive, Walden et al. found that the majority of injured players in their video analysis study landed either in heel strike or flat foot versus on the toe (18° vs. 9°, respectively) [29].

One PS study analyzed ankle plantarflexion in association with ACL injury predisposition [31]. They found no significant difference in ankle plantar flexion moment at initial contact or throughout the range of motion between female injury and non-injury participants [31]. Landing in a heel-strike position with lower degrees of dorsiflexion (i.e., more stiff) may contribute to an increased risk of ACL injury although the literature is limited and somewhat conflicting. 

*Trunk Angle and Lean:* One VA study [34] and four PS studies [11,16,19,28] accounted for trunk angle and lean as contributors to ACL injury (Table 5). Hewett et al. found that female basketball players had significantly higher mean lateral trunk angle at the time of injury than their male counterparts (mean 11.1° ± 2° vs. −5.5° ± 9.5° *p* = 0.04, respectfully) (Table 5) [16]. There was also a trend towards higher mean lateral trunk angle at injury between injured and non-injured female athletes [16]. In this study, injured female athletes also had less forward lean at the time of injury versus non-injured females (1.6° ± 9.3° versus 14.0° ± 7.3° *p* = 0.005, respectfully), but showed no difference from injured males (Table 5) [16]. Sheehan et al. found that injured athletes had positioned their center of mass (COM) more posterior than non-injured athletes at initial impact during one-legged landing maneuvers, indicating less forward trunk lean for injured subjects (Table 5) [28]. This finding was consistent between male and female athletes analyzed in this study. These studies suggest that landing with more upright posturing may increase ACL injury risk. 

Zazulak et al. found that trunk displacement in response to sudden unloading yields significant predictive value of future ACL injury [19]. Specifically, female injured athletes demonstrate higher peak trunk displacement and trunk displacement after force unloading versus injured male athletes and non-injured female athletes at initial contact. However, no significant differences were found for male injured or non-injured athletes. Similarly, in collegiate athletes, DuPrey et al. found a significant correlation between the time it takes athletes to stabilize their core following impact to ACL injury risk [34]. In their study, injured athletes had an average time to stabilization following a backward single legged jump of 0.49 s longer than non-injured athletes [34]. This trend was also observed for forward, medial, and lateral jumps, but was nonsignificant [34]. Dingenen et al. found an increase in combined dynamic knee valgus and lateral trunk motion in the direction of the stance limb during a pre-screened single-leg drop landing in ACL injured subjects [11]. These findings suggest that increased core stability and postural control may decreased ACL injury risk especially in female athletes.

## 4. Discussion

Despite the biomechanical, kinematic, and translational research on ACL injury risk factors, little consensus has been reached regarding the specifics of such factors and how they translate between the sexes. A recent systematic review found that females were at increased risk of non-contact ACL injury compared to male athletes, with the disparity being especially prevalent at the amateur level of competition [6]. Such injuries normally result in long absences from competitive sports and impose a high degree of financial burden on amateur athletes, many of whom are not receiving compensation or coverage for their participation in sporting events [37,38]. This finding furthers the need to better understand the factors contributing to ACL injury in order to better tailor training programs in the way of injury prevention and recovery [39,40]. 

Previous studies have highlighted the importance of malignant movement patterns regarding hip and pelvic positioning, knee angle, ankle positioning, and tibial rotation during certain athletic maneuvers [41,42]. Some have sought to attribute the disparity in relative risk between male and female athletes to hormonal differences [43,44,45] and neuromuscular control [20,46,47,48]. Yet, despite the clear association between female athletes and relatively higher risks of non-contact ACL injury in comparison to male athletes [6], specific malignant movement patterns have seldom been documented in the literature for those with confirmed ACL rupture. This review aimed not only to investigate biomechanical risk factors with high associations to ACL injury, but also to draw comparison between male- and female-specific risk factors, with the hope that future studies may be better aimed to expand our knowledge of how these variables play a role in certain individuals ACL injuries.

Hashemi et al. proposed that restricted hip flexion during landing may contribute to risk of ACL injury due to increased anterior tibial translation [49]. This increased tibial translation is likely the result of poor biomechanical advantage of the hamstrings with the knee in a more extended position, thus allowing increased forward motion of the tibia relative to the femur [21,23]. The increased anterior tibial translation contributes to escalating level of tension on the ACL complex which predisposes to higher incidence of rupture [50]. Subsequent studies have concluded that the hip musculature is critical for proximal control of the knee joint during athletic maneuvers [18,23,51]. Of the studies considered in this review that analyzed hip movement, the majority reported increased degree of hip flexion upon landing in injured athletes [25,26,28], and trends towards more hip abduction (Table 2) [25,29,35]. Multiple studies included in this review found injured athletes to have decreased hip mobility throughout load acceptance, higher degree of hip flexion at the end of loading and small changes in abduction versus uninjured athletes (Table 2) [25,26,31]. In contrast to mean hip flexion at initial contact, there was debate on whether peak hip flexion increased ACL injury risk (Table 2) [12,18,31]. While there was mixed evidence on the role of peak hip flexion motion on ACL injury risk, weaker hip abduction strength was found to be associated with increased ACL injury risk [17]. This is likely due to the increased hip adduction motion that results in dynamic valgus at the knee which predisposes the ACL to higher levels of shear force [15,18,52]. 

Despite the controversy on peak hip flexion, it is apparent that movement and strength at the hip contributes to ACL risk susceptibility. This may be a significant contributing factor to why females have a higher risk of non-contact ACL injury than males. In three studies reviewed, females not only had a propensity to land with higher degrees of hip flexion, but they also sustained their flexion angle longer compared to injured males and uninjured athletes [15,25,36]. This finding represents a significant and inherent difference in risk factors between male and female athletes and is an area that future research into ACL injury mechanisms should be focused. Additionally, females were found to have less hip abductor strength, by percentage of body weight, and delayed vastus medialis activation during a single leg drop jump than male athletes [17,36]. These findings may be due to inherent anatomical differences in the pelvic bone structure, differences in neuromuscular movement patterns, different training regimens, or a multitude of other reasons. Regardless of the origin, this difference seems to contribute to the increased relative risk female athletes face with ACL injuries. 

Knee joint kinematics and biomechanics have been widely studied in regard to the risk they impose on ACL injury. Previous studies have shown that both forces applied to extended knee joints [53,54,55] as well as high degree of knee valgus [12,13,14] has been linked to ACL injury. In this review, both knee flexion and knee abduction were considered. Results on knee flexion at initial contact were mixed with one reporting decreased [27], and four reporting no statistically significant association to ACL injury (Table 3) [18,25,32,33]. However, three studies reported increased peak degree of knee flexion (Table 3) [12,27,33], and one study reported progression towards higher levels of knee abduction during movement contributed to ACL injury [25]. This discrepancy in knee joint findings may have to do with the maneuver athletes were performing at the time of analysis and/or additional predisposing factors. Athletes landing with higher degree of hip flexion may have been predisposed to higher ACL strain and subsequent rupture, despite the angle of the knee, due to the hips’ role in knee stabilization [18,23,51]. Alternatively, movements with a high propensity to precipitate knee valgus could have a more significant impact on ACL injury than abduction and flexion, resulting in injury despite the sagittal angle. 

Previous studies have shown that increased transition to an abducted knee position during movement may predispose to ACL injury [25]. Additionally, others have shown female injured athletes had higher degree of knee abduction on landing than injured males [15,16]. This combination of findings helps to demonstrate the importance of longevity for female athletes. As athletes get older, it may be important to focus training programs on consistently maintaining safe habits of low degrees of knee abduction and landing in less stiff positioning during athletic maneuvers. This finding may also help to combat injuries at younger ages through early intervention [7,8]. In fact, one cohort study of over 1000 female athletes between the ages of 14 and 18 years old found that extensive neuromuscular and proprioceptive training reduced the two-year ACL injury risk by 74% [8]. Considering the potential benefit of injury prevention programs, training young female athletes in a way that combats increasing knee abduction with fatigue and age may decrease the propensity to develop such habits and may reduce lifetime risk of ACL injury. 

All studies reviewed in this paper considering ankle kinematics showed that landing with a heel strike (i.e., less plantarflexion on initial contact) was associated with ACL injury in athletes (Table 4) [26,27,28,31]. This pattern has been widely associated with the inability of the gastrocnemius to absorb the force when athletes land in a heel-strike pattern, resulting in the force being translated directly to the knee [25]. Additionally, one study found that injured athletes remained in a more plantarflexed position after initial contact for longer periods of time, while uninjured athletes more quickly transitioned to a dorsiflexed position [25]. This finding may further the idea that a lack of gastrocnemius ability to absorb landing force on ground strike contributes to ACL injury as it seems those that were injured had less movement around the ankle joint. In addition, less ankle motion likely results in less knee and hip motion during landing and predisposes these patients to landing in more stiff positioning. This absence of movement has been hypothesized to be a result of a lack of the lower kinematic chain to properly function, and thus further contributes to ACL injury [25]. 

Trunk stability is a relatively recent concept that some have suggested may contribute to safety and stability while performing a wide variety of athletic maneuvers. Specifically, it has been shown that trunk displacement can predict ACL injury risk with high degrees of specificity and sensitivity in female athletes [19]. However, this trend has not held up for male athletes [19]. This difference may be attributed to different hip-musculature activation patterns [56] and/or differences in interpretation of incoming sensory input contributing to bodily adjustments and knee stability [18,20,57,58] between men and women. All studies that considered trunk stability showed a propensity for female athletes to take longer to stabilize [34], hold their center of mass more posteriorly versus their base of support [16,28], and demonstrated higher mean lateral trunk angles (Table 5) [16,34] during athletic movements. Although these appeared to be significant contributors to female ACL injury, there was a significant difference from male counterparts in only two studies that found that females had higher mean lateral trunk angles [16,34] than their male counterparts. Nonetheless, core stability and trunk deviation are areas that may be improved with proper training [9,10,11]. These findings demonstrate the potential benefit of tailoring biomechanical training programs for athletes, in particularly female athletes, towards stability and agility in the way of injury prevention. 

Future studies on ACL injury mechanisms should seek to further elucidate the difference between male and female athlete injury risk factors. Larger-scale pre-screening studies may be beneficial to further understand biomechanical factors that predispose young athletes to ACL rupture. Ultimately, the implementation of a screening program for young athletes could be utilized to extrapolate patients into low, medium, and high-risk groups for ACL injury to guide need for early intervention and education on injury risk. Additionally, future studies should seek to analyze the benefit of strength training programs targeting known and suspected risk factors (i.e., higher degrees of hip flexion, landing in heel strike, trunk stabilization, etc.) to assess difference in relative risk of future ACL injury. 

## 5. Conclusions

The current literature is mixed in regard to biomechanical risk factors for ACL injury. However, some trends have emerged. In this review, we found that stiff landings, poor core stability, weak hip abduction strength, increased knee valgus, knee longevity over time, and landing in a heel strike position may increase the risk of ACL injury. All of these factors appear to significantly contribute to ACL injury, and are particularly prevalent in female athletes compared to males. This risk seems to be particularly pronounced in amateur female athletes. Young male and female athletes may benefit from targeted training programs, specifically those focusing on hip abductor strength, core stability, knee stability, and ankle positioning on landing for future injury prevention. Despite the wealth of knowledge about ACL injury risk, larger studies are needed to determine specific biomechanical factors predisposing patients to ACL injury. Future studies should also be directed at elucidating the benefits of targeted training programs to decrease the incidence of ACL injury in young athletes.

## Figures and Tables

**Table 1:** Demographic, Method, and Variable Breakdown by Studies Reviewed.

Primary Author	Study Quality	Female/Male	Sport	Time Span (Years)	Method	Analysis	Main Variable of Interest
**Video Analysis Studies**							
Koga 2010 [14]	VI	10/0	Basketball, Handball		case series	2D analysis	knee valgus & flexion, peak vertical ground-reaction force
Krosshaug 2007 [15]	VI	1/1	Basketball, Handball		cohort	3D analysis	knee valgus & flexion, peak vertical ground-reaction force, medial knee displacement
Hewett 2005 [18]	IV	205/0	Basketball, Soccer	1.5	cohort	3D analysis	knee valgus, ground reaction force, knee loading
Boden 2009 [25]	IV	33/23	Basketball, Soccer, Handball, Football, Gymnastics/Cheer		matched cohort	2D analysis	hip & knee motion
Koga 2018 [26]	VI	10/0	Basketball, Handball		case series	2D analysis	hip & ankle motion, COM
Montgomery 2018 [27]	IV	0/73	Rugby		matched cohort	2D analysis	ground contact angle, knee & ankle motion
Sheehan 2012 [28]	IV	26/14	Basketball, Soccer, Handball, Football		matched cohort	2D analysis	COM, limb angle, trunk angle
Walden 2015 [29]	VI	0/39	Soccer		case series	2D analysis	hip, knee, & ankle flexion
Olsen 2004 [30]	VI	52/0	Handball		case series	2D analysis	knee valgus & flexion
**Pre-Screen Studies**							
Dingenen 2015 [11]	IV	50/0	Soccer, Handball, Volleyball	1	cohort	2D analysis	hip flexion, knee valgus, lateral trunk motion
Krosshaug 2016 [12]	IV	710/0	Soccer, Handball	7	cohort	3D analysis	knee valgus, & flexion, vertical ground reaction force, medial knee displacement
Hewett 2009 [16]	IV	16/7	Basketball		matched cohort	2D analysis	trunk angle, knee valgus
Khayambashi 2015 [17]	IV	138/363	Football, Soccer, Volleyball, Basketball, Handball	1	cohort	Dynamometer	hip strength
Zazulak 2007 [19]	IV	140/137		3	cohort	Electromagnetic sensor	trunk displacement
Leppänen 2017 [31]	IV	171/0	Basketball, Floorball	3	cohort	3D analysis	hip, knee, & ankle motion
Leppänen 2017 [32]	IV	171/0	Basketball, Floorball	3	cohort	3D analysis	knee valgus & flexion, vertical ground-reaction force, medial knee displacement
Numata 2018 [33]	IV	291/0	Basketball, Handball	3	matched cohort	2D analysis	knee valgus
Duprey 2016 [34]	IV	112/166	Football, Volleyball, Field Hockey, Lacrosse, Basketball, Soccer	3.1	cohort	Force platform	TTS score

Table 1 displays the demographic data, timeline, method of analysis, and variables of interest for all studies considered in this review, when appropriate. Studies are described by primary author last name and publication date, and separated by video-analysis or pre-screen style of investigation. COM = center of mass, and TTS = time to stabilization (measure of postural stability).

**Table 2:** Hip Angle Variables Reported by Studies Reviewed.

	Hip Flexion_IC (Degrees)			Peak Hip Flexion Moment (N·m)			Hip Abduction (Degrees)		
	Injured	Control	*p*-Value	Injured	Control	*p*-Value	Injured	Control	*p*-Value
Hewett 2005 [18]	NS	NS		147.9 ± 33.5	106.8 ± 45.3	<0.01	NS	NS	
Boden 2009 [25]	50.1 ± 13.2	25.8 ± 14.7	0.0003	NS	NS		29.9 ± 11.0	25.7 ± 12.7	
Koga 2018 [26]	51			NS	NS		21	NS	
Montgomery 2018 [27]	26.5 ± 15.99 *	43.3 ± 24.8 *	0.26	NS	NS		NS	NS	
Sheehan 2012 [28]	48 ± 12	31 ± 22		NS	NS		NS	NS	
Walden 2015 [29]	15	NS		NS	NS		NS	NS	
Leppänen 2017 [31]	45.4 ± 10.7	43.5 ± 9.2	0.43	134.7 ± 42.4	122.9 ± 40.0	0.24	NS	NS	

Table 2 displays the mean hip angle/moment ± standard deviation (when available) of ACL injured vs. control subjects (those that were not injured). IC = initial contact; NS = not studied. * Data collected from direct communication with author.

**Table 3:** Knee Flexion Angle Values of Studies Reviewed.

	Knee Flexion IC (Degrees)			Peak Knee Flexion (Degrees)			Knee Abduction IC (Degrees)			Peak Knee Abduction Moment (N·m)		
	Injured	Control	*p*-Value	Injured	Control	*p*-Value	Injured	Control	*p*-Value	Injured	Control	*p*-Value
Krosshaug 2016 [12]	−2.2 ± 4.7	−1.7 ± 4.1	0.51	92.2 ± 13.8	90.8 ± 14.9	0.62	−2.2 ± 4.7	−1.7 ± 4.1	0.51	21.2 ± 12.2	20.9 ± 11.0	0.91
Hewett 2005 [18]				71.9 ± 12	82.4 ± 8.0	<0.05				−45.3 ± 28.5	−18.4 ± 15.6	<0.001
Boden 2009 [25]	21.8 ± 7.0	18.3 ± 7.5	0.2504	17.6	39.3	0.0001	5.5 ± 6.0	5.6 ± 6.7	0.96	NS	NS	
Montgomery 2018 [27]	13.6 ± 3.63 **	23.6 ± 17.3 **	<0.001 **	NS	NS		NS	NS		NS	NS	
Olsen 2004 [30]	15.8 ± 5.8	NS	NS	NS		13.15	NS		12.6	NS		
Leppänen 2017 [32]	30.2 ± 11.7	27.6 ± 9.0	0.29	81.5 ± 10.0	84.6 ± 10.3	0.25	0.9 ± 5.8	−1.8 ± 6.7	0.12	37.1 ± 24.9	31.2 ± 22.0	0.32
Numata 2018 [33]	NS	NS		NS	NS		2.1 ± 2.4 *	0.4 ± 2.2 *	0.006	8.3 ± 4.3	5.1 ± 4.1	0.007

Table 3 displays the mean knee flexion angle, reported as degrees ± standard deviation (when available) of ACL injured vs. control subjects (those that were not injured). IC = initial contact; NS = not studied. * reported in cm. ** Data collected from direct communication with author.

**Table 4:** Ankle Motion Values of Studies Reviewed.

	Ankle PF_IC (Degrees)		
	Injured	Control	*p*-Value
Boden 2009 [25]	10.7 ± 9.6	22.9 ± 10.1	0.0059
Koga 2018 [26]	−2.5 ± 18.6	NS	
Montgomery 2018 [27]	−2.5 ± 13.6 *	0 *	0.033 *
Leppänen 2017 [31]	7.4 ± 8.4	9.8 ± 9.6	0.26

Table 4 displays the values of mean measured ankle motion ± standard deviation (when available) of ACL injured vs. control subjects (those that were not injured). IC = initial contact; PF = plantarflexion; NS = not studied. * Data collected from direct communication with author.

**Table 5:** Trunk Angle and Motion Values of Studies Reviewed.

	Female Lateral Trunk Angle (Degrees)			Male Lateral Trunk Angle (Degrees)			Female Forward Trunk Lean (Degrees)		
	Injured	Control	*p*-Value	Injured	Control	*p*-Value	Injured	Control	*p*-Value
Hewett 2009 [16]	11.1 ± 2	4.2 ± 9.6	0.29	−5.5 ± 9.5	NS	0.04	1.6 ± 9.3	14.0 ± 7.3	<0.01
Sheehan 2012 [28]	4 ± 14	15 ± 13		6 ± 17	18 ± 14		NS	NS	

Table 5 displays the mean trunk motion ± standard deviation (when available) of ACL injured vs. control subjects (those that were not injured). NS = not studied.

## Data Availability

The data in this study are available on PubMed and other repositories.

## Supplementary Materials

The following are available online at https://www.mdpi.com/article/10.3390/ijerph18073826/s1. Figure S1: PRISMA 2009 Flow Diagram.
