# Supplementary material for: Stiff Landings, Core Stability, and Dynamic Knee Valgus: A Systematic Review on Documented Anterior Cruciate Ligament Ruptures in Male and Female Athletes
Source: Int J Environ Res Public Health. 2021 Apr 6;18(7):3826. doi: 10.3390/ijerph18073826 (PMC8038785; doi:10.3390/ijerph18073826)
Supplement: Supplementary file 1 [file ijerph-18-03826-s001.pdf]

PRISMA 2009 Flow Diagram

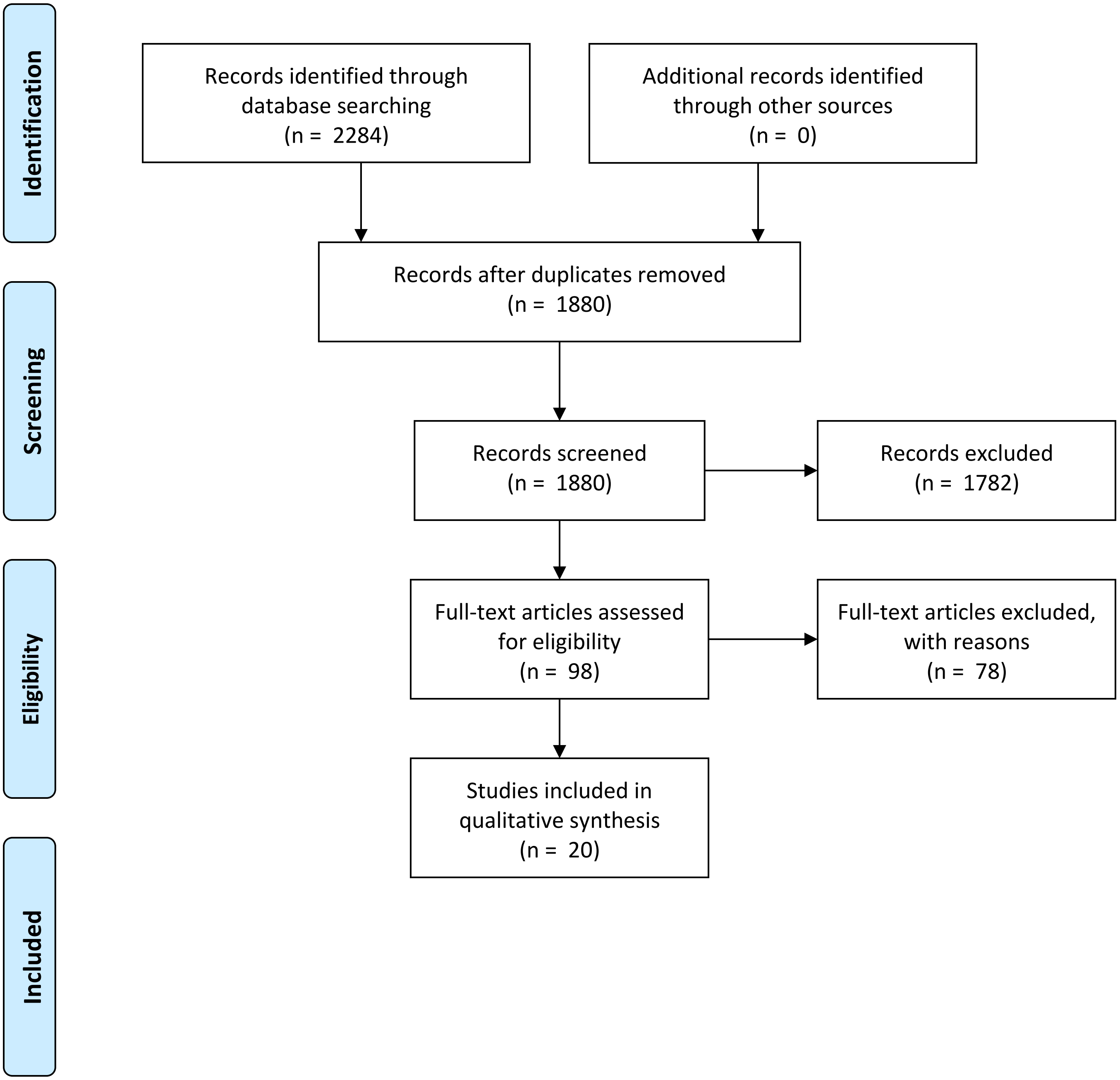

From: Moher D, Liberati A, Tetzlaff J, Altman DG, The PRISMA Group (2009). *P*referred *R*eporting *I*tems for *S*ystematic Reviews and *M*eta-*A*nalyses: The PRISMA Statement. PLoS Med 6(7): e1000097. doi:10.1371/journal.pmed1000097

For more information, visit www.prisma-statement.org.
